# Gene Therapy for the Treatment of Cardiac Arrhythmias: Current and Emerging Applications

**DOI:** 10.19102/icrm.2018.091204

**Published:** 2018-12-15

**Authors:** Amar Trivedi, Rishi Arora

**Affiliations:** ^1^Department of Cardiology, Northwestern Memorial Hospital, Chicago, IL, USA

**Keywords:** Atrial fibrillation, gene therapy, genomic therapy, ventricular tachycardia

## Abstract

In this review, we examine the current state of gene therapy for the treatment of cardiac arrhythmias. We describe advances and challenges in successfully creating and incorporating gene vectors into the myocardium. After summarizing the current scientific research in gene transfer technology, we then focus on the most promising areas of gene therapy at this time, which is the treatment of atrial fibrillation and ventricular tachyarrhythmias. We also review the scientific literature to determine how gene therapy could potentially be used to treat patients with cardiac arrhythmias.

## Introduction

Invasive cardiac electrophysiology is a relatively young field in medicine. Modern ablative tools and techniques were developed and refined only in the last 50 years.^1^ However, the treatment of cardiac arrhythmias is still suboptimal. Many diseases such as atrial fibrillation (AF) and ventricular tachycardia (VT) are difficult to cure with either invasive or pharmacological therapy.^2^ Given the challenges of treating patients with heart rhythm disorders, scientists are actively investigating new treatment paradigms such as the use of gene therapy to treat cardiac arrhythmias. The field of gene therapy holds great promise for creating highly effective, personalized treatments for patients with cardiac arrhythmias. In this review, we begin by discussing the current state and advances in gene transfer technology and then examine that status of gene therapy for AF as well as ventricular arrhythmias, in an effort to elucidate the potential direction(s) that the research and application of genomics in cardiac arrhythmia management may take.

## The basic components of myocardial gene transfer

For any potential cardiac gene therapy to be successful, it must be trafficked to the myocardium and expressed at appropriate concentrations in the cardiac chamber(s) of interest. This is a two-step process in which (1) a vector is created to house the gene of interest and then (2) the gene is delivered to the myocardium. There are two types of vectors, nonviral and viral vectors, which are discussed henceforth.

### Nonviral gene vectors

The predominant nonviral gene vector is naked plasmid deoxyribonucleic acid (DNA) directly injected into the myocardium via a process called transfection. Transfection is the incorporation of nonhost DNA via mechanical disruption of the host’s cellular membrane integrity. Plasmid DNA has been used with success in cardiac gene trials and is usually transduced to the cell via the process of lipid-mediated transfection.^3^ The benefits of plasmid DNA administration include scalability, limited cellular and antibody-mediated immune response, and the ability to store a large DNA library with ease. A key advantage of plasmid vectors in comparison with other types of vectors is the lack of an antibody-mediated immune response. This allows therapies to be administered multiple times without resulting in adverse immune reactions.

The most vexing problem to the widespread adoption of plasmid vectors is the low transfection rate when delivering genetic material.^4^ Current lipid-mediated transfection technology only allows for a negligible amount of gene to enter the myocardium.^5^ Complexing agents, which are biochemical molecules such as cationic lopisomes or chemicals such as calcium phosphate, can be added to naked plasmid DNA to facilitate cellular uptake.^6^ These have been shown to marginally improve the efficiency of gene uptake, but their impact remains limited overall.^6^ A promising method of increasing drug delivery is sonoporation. Using ultrasound technology, energy can be noninvasively transmitted to a variety of structures throughout the myocardium. Genes can be placed in microbubbles that “burst” with the application of ultrasound energy when the microbubbles pass through the target of interest. In the myocardium, both naked plasmid DNA and short interfering ribonucleic acid (siRNA) have been transduced into the adult murine heart via sonoporation.^7^

Our group used nonviral DNA vectors with an alternative method to efficiently deliver gene therapy into the canine myocardium.^8^ Using a process known as electroporation-guided gene delivery, we can safely introduce high levels of gene therapies in specific locations. Electroporation is a two-step process that involves the injection of a nonviral gene vector into the myocardium, followed by the performance of synchronized pulses over short cycles of time, which drives the target gene into the tissue.^8^ The rate of gene uptake is 15- to 20-fold higher when electroporation is used as compared with the rate seen with standard plasmid DNA delivery.^9^ Electroporation is the most effective and well-developed method of nonviral gene vector delivery to date. Its advantages include a relative ease of use, low equipment cost, and excellent ability to successfully transfer gene therapy throughout the myocardium. The main disadvantage is the need for, at minimum, a minithoractomy to visualize the epicardium and to effectively deliver gene therapy and electricity to sites of interest. Electroporation of nonviral vectors represents an exciting platform that can potentially allow for the safe delivery of gene therapy to the human myocardium.

### Viral gene vectors

In contrast, viral vectors are live viruses that have been genetically modified to allow the insertion of transgenes to occur without the wild-type viruses maintaining their ability to reproduce or cause morbidity. As compared with nonviral plasmid insertion, which must be directly injected into the tissue, viral vectors have the theoretical advantage of minimally invasive delivery via the bloodstream through a process called transduction. Unlike plasmid DNA transfection, which requires mechanical disruption of the membrane, transduction allows for genetic transfer by the way of viral vectors overtaking normal cellular machinery.

The most commonly used viral vector is the retroviral vector. This has been used in numerous United States Food and Drug Administration–approved clinical studies in both phase I and phase II trials including regarding the treatment of glioma and severe combined immunodeficiency. These studies have raised safety concerns about the risks of retroviral therapy, but advances in retroviral vector technology hope to mitigate such.^10^ However, the use of retroviral vectors in the myocardium is limited. Retroviral vector technology requires active cellular reproduction to allow for the integration and expression of its transgene. This is problematic in terminally differentiated tissues such as cardiac myocytes. To overcome these obstacles, an area of active investigation is the use of lentiviral vectors, in which the human immunodeficiency virus machinery is used to transfer gene therapy to postmitotic cells such as cardiac myocytes.^11^ Lentiviral vectors can transfect intact nuclear membranes, allowing for transgene expression to occur in terminally differentiated cells such as cardiomyoctes. A major advantage of lentiviral technology is what appears to be long-term gene expression and multiple safeguards to protect against wild-type reversion as compared with in the case of other retroviral vectors.^12^ Lentiviral vectors have been also been compared with nonretroviral vectors, such as adenovirus (AD) vectors, and appear to have similar efficiency when injected into the myocardium.^11^ However, their safety and efficacy have yet to be demonstrated in any clinical trial of cardiac gene therapy to date.

ADs and adeno-associated viruses (AAVs) are currently the most commonly used viral vectors for cardiac gene therapy transduction. The wild-type AD is a double-stranded DNA virus that is one cause of the common cold. AD vectors have the viral genome (35 kb) removed to allow for the delivery of moderately sized genes. This leads AD vectors to be simple to produce and distribute and to be transduced with relative effeciency.^13^ However, the AD vector has limited gene expression (ie, two weeks to four weeks) and can cause intense immunological host responses that may lead to short-term morbidity and organ damage.^14^ This has led to disappointing short-term results for the AD vector in clinical trials.^15,16^

These limitations have led to the development of AAV vectors, which share no relationship to ADs and are a distinct class of virus. These vectors have multiple advantageous features including the possibility of long-term gene expression. In skeletal muscle, gene expression with AAV vectors has persisted for several years in some preclinical trials.^17,18^ The disadvantages of AAV vectors include a limited gene insert size, which makes it difficult to transduce larger genes and complex ion channels, as well as the existence of delayed expression of the gene, likely due to the need to convert the single-stranded viral genome to the double-stranded host genome.^19^ Another limitation is that the transduction efficiency is more limited in larger mammal studies versus in the initial studies in rodents.^20^

## Gene delivery to the myocardium

Once a vector is chosen, the next challenge is choosing the optimal method to deliver the vector to the target organ. To affect a wide variety of arrhythmia mechanisms, gene therapy should provide dense, transmural, and homogenous expression in the myocardial chamber(s) of interest. To date, the ability to provide a delivery system that accomplishes the above goals remains one of the greatest challenges in translating molecular discoveries to actionable clinical therapies. At this time, there are three widely described methods to deliver gene vectors (both viral and nonviral) to myocardial tissue: direct injection, intracoronary perfusion, and epicardial gene painting.

Of the three methods described above, gene injection is the most simple and well-studied one. It involves direct injection of the gene vector into myocardial tissue. The vector can be injected either epicardially or endocardially and produces a very high concentration of gene expression at the site of injection. However, gene expression only occurs within a few millimeters of the injection site, making delivery to a large area of the myocardium technically challenging.^21^ The use of electroporation as discussed previously improved the efficiency and depth of gene transfer, but direct injection is still not a practical option to treat multiple chambers of the heart.

One method to overcome this limitation is delivering genetic vectors to the coronary vasculature, allowing for the entire heart or selected regions of the left ventricle to be targeted. Intracoronary delivery can be used to deliver gene vectors via the coronary arteries.^22,23^ However, the conditions needed to optimize vector transduction are difficult to perform in humans. They include hypothermia to 18°C; disruption of normal coronary blood flow; and high doses of nitroglycerin, adenosine, and vascular endothelial growth factor. With optimization, intracoronary AD vectors have been successfully delivered to the anteroseptum of the left ventricle with high levels of gene expression.^24^ The safety of intracoronary infusion was demonstrated in both the Calcium Upregulation by Percutaneous Administration of Gene Therapy in Cardiac Disease (CUPID) and CUPID 2 trials. In these trials, an AD-associated vector carrying the *SERCA2a* gene was inserted in patients with systolic heart failure via intracoronary perfusion. *SERCA2a* is a sarcoplasmic reticulum calcium adenosine triphosphate (ATP)-ase that is deficient in patients with advanced heart failure.^25,26^ Both trials met their primary safety endpoints and demonstrated that gene therapy can be safely administered in patients with advanced heart failure.

Another approach to successfully deliver vectors to both atria in a transmural fashion is the process of epicardial gene painting, which was first employed by Kikuchi et al.^27^ This technique involves the application of a combination of a gene vector, a poloxamer compound, and a protease (trypsin) to the atrial epicardium. The poloxamer compound improves vector contact with the atria. Trypsin is used to foster transmural penetration of the vectors. This method has been demonstrated to be safe and effective in animals models with minimal to no impact on atrial structure or function.^24,27^ Epicardial gene painting has come closest to allowing a homogenous transmural application of gene therapy, but is somewhat limited because it requires an epicardial approach, necessitating a surgical procedure. It is also unclear as to whether this technique would also lead to transmural gene delivery in the ventricle as well or not.

## Gene therapy for atrial fibrillation

Of all the potential cardiac arrhythmias to be treated with gene therapy, AF has been the most intensely researched. The development of AF increases a patient’s risk of stroke, heart failure, dementia, and death.^28,29^ There are many distinct genetic loci associated with AF identified through genome-wide association studies.^30^ There also appears to be other loci that have been found through familial linkage studies.^31^ One of the most challenging aspects of AF treatment is the heterogeneous genetic, structural, and electrical abnormalities that lead to the condition. Our current treatment strategies for restoring normal sinus rhythm in AF are limited by suboptimal efficacy and potential morbidity and mortality. Pharmacological therapy with antiarrhythmic drugs can have long-term drug side effects.^32,33^ AF ablation is increasingly being used to target patients with symptomatic AF but has limited efficacy as well as significant morbidity including risk of death.^34,35^ In the following discussion, we highlight some of the more promising gene therapy approaches that exist for preventing and treating AF.

### Targeting ion channels

In AF, a common mechanism of electrical remodeling is the shortening of the action potential duration (APD). By shortening the APD, reentrant circuits are more easily inducible and maintained. Gene therapy has been used to prolong the APD in animal models by reducing the expression of the delayed rectifier potassium channel *I*_Kr_. This occurs by the inhibition of the *KCNH2* gene, which is responsible for the alpha subunit of the *I*_Kr_ channel. Amit et al.^36^ demonstrated in a pig model that APD could be prolonged via the epicardial gene painting of an AD vector encoding a dominant negative mutation of *KCNH2*. By interrupting the alpha subunit of the *I*_Kr_ channel in the gene-treated pigs, the investigators found an increase in the APD, resistance to burst pacing-induced AF, and increased conversion from AF to sinus rhythm. These effects were reversed at two weeks, which correlated with a loss of gene expression. In another proof-of-concept study, Soucek et al.^37^ used epicardial injection and electroporation to deliver the AdCERG-G627S transgene to disrupt *KCNH2* function. They found that pigs who received gene therapy had markedly longer APD and that the development of persistent AF was delayed or prevented in a rapid atrial pacing model. Furthermore, when compared with the control group, the pigs that received the transgene and developed persistent AF did not develop impaired left ventricular function. Notably, however, both studies were of too short a duration to determine the long-term side effects of atrial *I*_Kr_ suppression.

### Repairing dysregulated gap junctions

Gap junctions are key regulators of electrical conduction in the atria. Gap junctions regulate intracellular conduction velocity by connecting the cytoplasm of adjacent cells. In human atria, there are two connexin subunits that form gap junctions: CX40 and CX43. Both animal models and human atrial tissue research have demonstrated that the decreased expression of these two connexins leads to AF-associated remodeling. Bikou et al.^38^ demonstrated that, in pigs, CX43 expression can be restored via epicardial direct injection/electroporation of an AD-encoding CX43. They found that gene-treated pigs did not develop AF over 14 days of rapid atrial pacing, while all of the control pigs developed AF. Another study in which epicardial painting restored the expression of both connexins in a rapid atrial pacing pig model showed improved conduction parameters with enhanced gap junction concentration.^39^

### Modifying the structural aspects of the atrial fibrillation substrate

Basic molecular investigations have shown that AF is associated with an underlying proinflammatory state, which leads to significant oxidative stress on the atria. This increase in inflammation and oxidative stress creates an imbalance in regulatory mechanisms, resulting in increased cellular fibrosis and apoptosis. Extensive work in multiple organ systems has shown that organ fibrosis is intimately correlated with the upregulation of transforming growth factor beta (TGF-β).^40,41^ TGF-β stimulates the production of collagen and other extracellular matrix proteins as well as the generation of reactive oxygen species.^41^ In the myocardium, the posterior left atrium has been found to have a unique role in the maintenance of AF due to an increased susceptibility to fibrosis and heterogeneous conduction.^42^ Our group has previously demonstrated that transduction of a transgene that interferes with TGF-β signaling in the posterior left atrium can profoundly change the structure and electrophysiology of such.^42^ Using direct injection and electroporation, a potent dominant negative mutation of the TGF-β receptor was transduced to the posterior left atrium of 12 canines. Reverse remodeling of the posterior left atrium improved conduction, and a reduction in pacing-induced AF was seen after three to four weeks of rapid pacing. There was also a change in the restitution slope, making the plasmid-injected atrial tissue more resistant to AF. These electrical changes were correlated with reduced atrial fibrosis, demonstrating a link between the AF substrate and conduction properties. These findings point to the ability of gene therapy to affect both the structure and function of atrial tissue by downregulating the inflammatory response seen in AF. This mechanistic insight may be useful not only for treating but also preventing the structural changes that foster the development of AF.

The increase in cellular apoptosis is another potential target for gene therapy. Dysregulation of the superoxide dismutase 1 (SOD1) enzyme occurs in a canine AF model. SOD1 plays an important role in cardiac apoptosis as well as oxidative stress signaling.^43^ Zhang et al. demonstrated that silencing micro-RNA (miR) 206 could decrease susceptibility to AF by reducing the activity of SOD1. They transduced an anti-miR-206 lentivirus into the superior left gangiolonated plexi in canines. These canines demonstrated a reduction in AF inducibility as well as a prolonged APD directly related to the effects of the anti-miR-206 lentivirus on the SOD1 enzyme. Another method of targeting apoptosis is through knocking down the activity of caspase 3, a key apoptotic enzyme that can be inhibited with siRNA. In a porcine model, treatment with an AD vector containing siRNA that targeted caspace 3 led to a suppression of apoptotic activity within the atrium as well as a delayed onset of persistent AF.^44^

## Gene therapy for the treatment of ventricular arrhythmias

Sudden cardiac death as the result of ventricular tachyarrhythmias is a leading cause of death worldwide.^45^ Ventricular arrhythmias represent a heterogeneous group of disorders that can be broadly classified into congenital disorders that can globally affect the ventricular myocardium and ischemia-driven arrhythmias secondary to ischemia and scar.

### Inherited cardiac channelopathies

Catecholaminergic polymorphic VT (CPVT) is an inherited arrhythmia disorder in which exercise and emotional stress can cause life-threatening polymorphic VT. A common gene mutation in CPVT involves the membrane-binding protein calseqsterin (CASQ2), which is localized to the sarcoplasmic reticulum and is crucial to the proper calcium storage and regulation of APD.^46^ In CPVT, this dysregulation in calcium handling causes delayed afterdepolarization as well as triggers activity due the diastolic release of calcium. To understand if gene transfer could improve calcium regulation, Denegri et al.^47^ developed a mouse model of CPVT by creating a knock-in mutation that disables *CASQ2* gene expression. They then engineered an AAV vector containing the wild-type *CASQ2* gene and transfected the vector to the CPVT model mice. The investigators subsequently found that the mice that received the wild-type *CASQ2* gene experienced a restoration of *CASQ2* expression, a normalization of electrophysiological and structural abnormalities, and a return of calcium handling to baseline. These results lasted for one year, and the gene-transfer mice demonstrated a marked suppression in bidirectional VT throughout the experiment.

### Ventricular arrhythmias caused by acute ischemia

In the setting of an acute myocardial infarction, reperfusion of ischemic cardiac tissue leads to a marked increase in the intracellular sodium and calcium concentrations. The increased concentrations are thought to be the driver of VT and ventricular fibrillation (VF) in the setting of an acute infarct.^48^ The role of calcium handling in acute ischemia has been well-studied, and calcium dysregulation occurs when ischemia leads to a reduction in intracellular ATP levels. This creates a cascade of cellular messaging activity, which decreases the activity of calcium pumps. In the sarcoplasmic reticulum, the ATP-dependent calcium pump *SERCA2a* is critical for intracellular calcium homeostasis and is dowregulated in acute ischemia. This leads to increased diastolic calcium release, which can cause triggered activity and delayed afterdepolarizations, leading to VT and VF. Gene transfer of wild-type *SERCA2a* via an AD vector has been shown to improve calcium in multiple animal models.^49^ In a pig model, for example, direct injection of an AD vector of *SERCA2a* into ventricular myocardium resulted in a significant reduction of VT/VF as compared with in control subjects.^50^ In other studies, *SERCA2a* gene transfer also appeared to improve resistance to pacing-induced VT in pigs with well-healed myocardial infarctions.^51^

### Ventricular arrhythmias caused by scar

Ventricular arrhythmias caused by myocardial scaring are commonly the result of reentry around or within areas of the scar. This can be due to previous episodes of ischemia or other myopathic processes that remodel the left ventricle. Reentrant circuits require heterogeneous and slow conduction as well as shortened refractory periods to propagate and maintain VT. This is especially the case at the border zone between scar and healthy tissue, where ion channel physiology is often impaired. Similar to the changes seen in the atria, in the setting of AF, gap junction expression is downregulated after a myocardial infarction.^52^ The expression of connexin CX43 is reduced in the ventricular myocardium after an instance of myocardial infarction. Similar to gene therapy transfer for AF, the use of an AD vector containing the *CX43* gene has been studied in an animal model of a healed myocardial infaction.^53^ Four weeks after a myocardial infarction, pigs were injected with an AD containing the *CX43* gene. The animals that received the gene demonstrated a marked reduction in inducible VT manifested as improved conduction parameters and a prolongation of the ventricular effective refractory period.^54^

## Future outlook

Gene therapy for the treatment of cardiac arrhythmias is still largely in the proof-of-concept stage. However, early results in animal models have been promising, as discussed in this review. As our molecular understanding of cardiac arrhythmias advances, our targets for gene therapy are expected to become more precise. New genomic technologies such as CRISPR/Cas9 editing hold promise for creating more accurate models and new avenues for therapeutic research.^55^ In the meantime, as electrophysiologists continue to utilize all areas of scientific research to search for more effective treatments of cardiac arrhythmias, gene therapy may provide an avenue to help better comprehend, if not treat, those suffering from these conditions.
